# Severe 25-Hydroxyvitamin D Deficiency May Predict Poor Renal Outcomes in Patients With Biopsy-Proven Diabetic Nephropathy

**DOI:** 10.3389/fendo.2022.871571

**Published:** 2022-05-04

**Authors:** Ting Zhou, Li Shen, Ze Li, Junjie Jia, Haifan Xing, Niansong Wang, Qiong Jiao, Ying Fan

**Affiliations:** ^1^ Department of Nephrology, Shanghai Jiao Tong University Affiliated Sixth People’s Hospital, Shanghai, China; ^2^ General Practice Department, The First Affiliated Hospital of Nanjing Medical University, Nanjing, China; ^3^ Clinical Research Unit, Shanghai Jiao Tong University Affiliated Sixth People’s Hospital, Shanghai, China; ^4^ Department of Pathology, Shanghai Jiao Tong University Affiliated Sixth People’s Hospital, Shanghai, China

**Keywords:** 25(OH)D, diabetic nephropathy, renal biopsy, prediction, pathology

## Abstract

**Aims:**

This study aims to investigate the role of 25-hydroxyvitamin D (25(OH)D) levels in predicting renal survival in biopsy-proven diabetic nephropathy (DN) with type 2 diabetes mellitus (DM).

**Methods:**

In this retrospective study, a total of 161 biopsy-proven DN patients were enrolled and divided into four groups (normal group: 25(OH)D>20ng/ml; mild group: 10<25(OH)D ≤ 20ng/ml; moderate group: 5<25(OH)D ≤ 10 ng/ml; severe group: 25(OH)D ≤ 5 ng/ml). The effect of the 25(OH)D level on renal survival was evaluated by multivariate Cox regression.

**Results:**

A total of 161 type 2 DM patients with biopsy-proven DN were enrolled in this study. Patients with lower 25(OH)D levels had higher serum creatinine, urinary albumin creatinine ratio (UACR), total cholesterol, and parathyroid hormone levels as well as lower estimated glomerular filtration rate (eGFR), hemoglobin, albumin, and calcium levels and were more prone to diabetic retinopathy (DR). Rather than proteinuria and renal function, glomerular class and interstitial fibrosis and tubular atrophy (IFTA) had a significant correlation with 25(OH)D levels. Multivariate Cox regression indicated that severe deficiency of 25(OH)D levels was associated with adverse renal outcomes. Compared to the level in the normal group, after adjusting for clinicopathological characteristics, a lower 25(OH)D level remained a risk factor for renal outcomes. The HRs were 3.446 (95% CI 0.366-32.406, p=0.279) for the mild group, 8.009 (95% CI 0.791-81.102, p=0.078) for the moderate group, and 14.957(95%CI 1.364-163.995, P=0.027) for the severe group.

**Conclusion:**

Levels of 25(OH)D less than 5 ng/ml were correlated with worse renal function, more pathological injury and poorer renal prognosis in patients with biopsy-proven DN.

## Introduction

Diabetes mellitus (DM) is a key public health problem worldwide, predicted to affect more than 700 million patients by 2045 (1).As one of the most common microvascular complications of DM, diabetic nephropathy (DN) has become a primary cause of end-stage renal disease (ESRD), and approximately 40% of hemodialysis patients have DN (2–4). DN has an insidious onset, lacks typical clinical symptoms and progresses slowly in the early stage, which is easy to ignore. Once DN progresses to ESRD, it seriously affects the quality of life and safety of patients. Although there are many hypoglycemic drugs with renal protective effects, the risk of DN progressing to ESRD and the economic burden of DN are still increasing (5). Therefore, it is urgent to further understand the pathogenesis of DN and the risk factors affecting the progression of DN for promoting clinical diagnosis and treatment.

Vitamin D, a fat-soluble vitamin essential for human growth, mainly binds with intracellular specific Vitamin D receptor (VDR) to form hormone receptor complex and play a biological role. As the main target organ of Vitamin D, VDR is selectively expressed in glomerular mesangial cells, podocytes, proximal and distal convoluted tubules, collecting tubules and other renal structures (6). The protective effect of Vitamin D and VDR in DN has attracted wide attention. Low level of Vitamin D has been found to be negatively correlated with the risk of DN (7). Supplementation of Vitamin D or its active analogues can improve endothelial cell damage, reduce proteinuria, and alleviate renal fibrosis, thus delaying the progression of DN (6, 8). Animal studies showed that active vitamin D can reduce the synthesis of renin and inhibit renin angiotensin aldosterone system (RAAS) to protect kidney from injury *via* inhibiting nuclear factor-kappa B (NF-κB) mediated anti-inflammation pathway (6, 9).

Prior studies have depended on the measurement of serum 25-hydroxyvitamin D (25(OH)D) when evaluating the relationships of vitamin D status with health outcomes. 25(OH)D is thought to be a stored and inactive form of vitamin D in the human body. 25(OH)D deficiency is common in all subsequent stages of chronic kidney disease (CKD), including ESRD (10). Lower levels of 25(OH)D in patients with CKD have been associated with a higher risk of all-cause mortality and faster progression of kidney disease (11). In previous studies of patients with CKD due to any cause, lower 25(OH)D levels were associated with an increased risk of incident ESRD (12) and contributed to decreased estimated glomerular filtration rate (eGFR) in early and advanced stages of CKD (13). However, few studies have explored the role of 25(OH)D in evaluating disease severity or progression in DN, and the relationship between 25(OH)D and the long-term prognosis of patients with DN is still unclear.

Therefore, in the current study we examined whether 25(OH)D deficiency was an indicator that not only reflects disease severity and pathological injury in DN, but also predicts disease prognosis in patients with biopsy-proven DN. This new finding will improve the management and renal outcomes of DN patients.

## Materials and Methods

### Study Design and Patients

**Figure 1:**
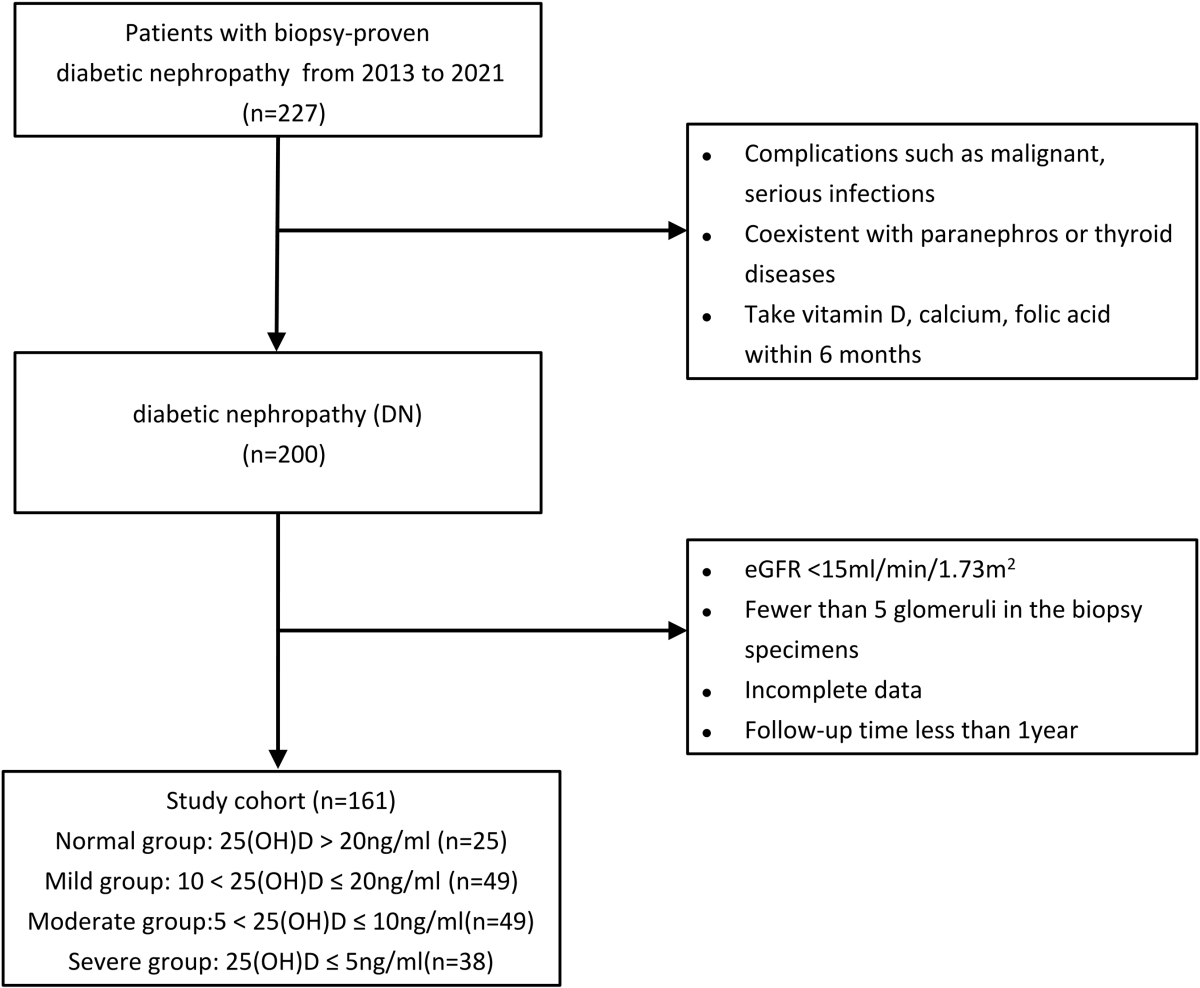
Study participant flowchart. eGFR, estimated glomerular filtration rate; 25(OH)D, 25-hydroxyvitamin D.

This is a retrospective cohort study comprised of 227 patients with biopsy-proven DN and type 2 DM at Shanghai Sixth People’s Hospital from January 2013 to October 2021. 161 of 227 type 2 DN cases were finally eligible for the enrollment (**Figure 1**). We divided the 161 patients into four groups on the basis of 25(OH)D level [normal group: 25(OH)D > 20 ng/ml; mild group: 10 < 25(OH)D ≤ 20 ng/ml; moderate group: 5 < 25(OH)D ≤ 10 ng/ml; and severe group: 25(OH)D ≤ 5 ng/ml] according to the cutoff value of renal endpoint events and some authoritative studies (14, 15). The diagnosis of type 2 DM was in accordance with the criteria of the American Diabetes Association (16), while DN was defined according to the standard of the Renal Pathology Society (RPS) (17) and diabetic retinopathy (DR) was diagnosed if one of the following lesions was observed: soft/hard exudates, retinal hemorrhage, microaneurysms, or vitreous hemorrhage (18). All patients with type 2 DM and biopsy-proven DN who were over 18 years old were reviewed. The exclusion criteria were patients with coexisting nondiabetic renal disease (NDRD) and paranephros or thyroid diseases combined with malignancy and serious infections and those who had taken vitamin D, calcium, or folic acid within 6 months. Patients who had incomplete data or unclear medical history, follow-up time less than 1 year, less than 5 glomeruli in the biopsy specimens, or eGFR < 15ml/min/1.73 m^2^ were also excluded, as were patients who had undergone dialysis or renal transplant before renal biopsy.

The study protocol was reviewed and approved by the Ethics Committee of the Shanghai Jiao Tong University Affiliated Sixth People’s Hospital and was in full accordance with the Declaration of Helsinki. All patients included in this study provided signed informed consent (NO. 2016-41).

### Clinical and Pathological Data

All data was collected from patients record files. Baseline clinical data were collected at the time of biopsy, including age, gender, presence of hypertension, systolic pressure, diastolic pressure, course of diabetes, presence of DR, and use of RAAS inhibitors. Serum albumin, total cholesterol, triglycerides, serum calcium, serum phosphorus, hemoglobin were measured *via* an automated analyzer using standard methods. Glycosylated hemoglobin A1c was measured using the Variant II Hemoglobin Analyzer of Bio-Rad Company in the United States (High Performance Liquid Chromatography). 25(OH)D, serum creatinine, urinary creatinine, urinary albumin, serum uric acid and parathyroid hormone (PTH) were determined by electrochemiluminescence (Roche, Germany, COBAS e601). Urinary albumin creatinine ratio (UACR) is calculated from urine albumin and urine creatinine. eGFR was calculated according to the CKD-EPI formula of the American Chronic Kidney Disease Epidemiology Collaborative Working Group. All biopsy samples were independently reviewed by two different pathologists. The pathological classification of DN was based on the RPS proposed in 2010 (17).

### Renal Biopsy and Follow-up

All patients enrolled in this study had undergone ultrasound-guided percutaneous renal puncture when they were in the hospital, and the biopsy indications were as follows (19, 20): 1) sudden onset of overt proteinuria; 2) the presence of glomerular hematuria; 3) a rapid decline in eGFR over a 3-month period; 4) no diabetic nephropathy; and 5) renal function decreasing rapidly with a short course of DM (<5 years). All biopsy specimens were cut into 3 μm sections, which were stained with Masson’s trichrome, periodic acid–Schiff and hematoxylin and eosin (H&E).

The average follow-up time was 38 months. Renal endpoint events were defined as a twofold increase in creatinine, ESRD (eGFR < 15ml/min/1.73 m^2^ or first onset of dialysis) and death.

### Statistical Analysis

At present, there is no unified international grading standard for 25(OH)D levels. However, in the literatures that have been published, they put the cut-off value for mild 25(OH)D deficiency between 10-20ng/ml (14, 15, 21). Based on comprehensive literature reports, combined with measurement methods of our hospital, we defined 25(OH)D>20ng/ml as normal value and 10-20ng/ml as mild deficiency. In addition, the area under the receiver operating characteristic curve (AUC) was analyzed by using logistic regression to identify the relationship between 25(OH)D and renal endpoints, the cutoff value was 5ng/ml. Given that, the patients in this study were divided into 4 groups according to the level of 25(OH)D: normal group(<20ng/ml), mild group(10-20ng/ml), moderate group(5-10ng/ml) and severs group(<5ng/ml).

SPSS software (version 21.0; SPSS Inc., IL, USA) was used for analysis of all data. For continuous variables, those with a normal and skewed distribution are described using the mean and standard deviation (SD) and median and interquartile ranges (Q1-Q3), respectively. Categorical variables are presented as frequencies (percentages). Differences in means for quantitative variables were evaluated using ANOVA or the Kruskal-Wallis H test, as appropriate. Categorical variables were compared with the chi-squared test. Correlations of the 25(OH)D level with clinicopathological findings were analyzed by Spearman’s correlation analysis and Kendall’s tau-b analysis. Kaplan–Meier curves were used to analyze cumulative renal survival, with comparisons between groups being made by the log-rank test. Cox analysis was used to explore the relationships between 25(OH)D levels and renal outcomes. The 25(OH)D level was first calculated as a continuous variable with hazard ratios (HRs) that resulted from each SD increment, and the different 25(OH)D groups were used as categorical variables, with the normal group regarded as a reference. A P value<0.05 was considered indicative of a significant difference, and the Holm-Bonferroni method was applied to reduce the risk of a type 1 statistical error.

## Results

### Baseline Clinical Characteristics

A total of 161 type 2 DM patients with biopsy-proven DN were enrolled in this study. The mean age was 53.48 years old at the time of renal biopsy. The median duration of the diabetes course was 10 years, and the mean HbA1c was 7.87%. The mean eGFR was 68.97 ml/min/1.73 m^2^, the median serum creatinine was 107 µmol/l, the median UACR was 2.58 g/d, and the mean serum calcium was 2.22 mmol/l at baseline. Patients with lower 25(OH)D levels had higher serum creatinine, UACR, total cholesterol, and PTH levels as well as lower eGFR, hemoglobin, albumin, and calcium levels and were more prone to DR (shown in **Table 1**). The average follow-up time was 38 months, and a total of 42 patients progressed to renal endpoints during follow-up.

**Table 1:** Baseline clinical characteristics in the groups stratified according to the 25-hydroxyvitamin D level.

Variables	Normal group >20ng/ml (n = 25)	Mild group 10-20ng/ml (n = 49)	Moderate group 5-10ng/ml (n = 49)	Severe group <5ng/ml (n = 38)	P value
Demographic features					
Age (year)	52.64 ± 9.21	56.94 ± 8.57	54.16 ± 9.94	50.19 ± 10.35	0.013
Men (%)	21 (84.0%)	32 (65.3%)	37 (75.5%)	25 (65.8%)	0.281
Course of diabetes (year)	8.00 (3.00-12.00)	10.00 (7.00-15.50)	10.00 (5.00-18.50)	10.00 (5.00-12.5)	0.287
Systolic pressure (mmHg)	133.20 ± 20.83	137.24 ± 19.94	144.20 ± 21.81	144.21 ± 19.51	0.070
Diastolic pressure (mmHg)	83.12 ± 10.84	80.51 ± 9.92	80.94 ± 11.74	83.68 ± 9.55	0.451
Hypertension (%)	10 (40.0%)	22 (44.9%)	28 (57.1%)	26 (68.4%)	0.074
Diabetic retinopathy (%)	5 (20.0%)	26 (53.1%)^†^	28 (57.1%)^†^	35 (92.1%) ^†‡#^	<0.001
Use of RAAS (%)	20 (80.0%)	38 (77.6%)	43 (89.6%)	28 (73.7%)	0.238
Biochemical findings					
HbA1c (%)	7.45 ± 1.56	7.95 ± 1.52	7.84 ± 1.57	8.26 ± 2.01	0.317
Serum creatinine (umol/L)	82.00 (64.50-133.00)	87.00 (62.00-106.50)	121.00 (91.00-162.00)^‡^	126.50 (108.25-197.25)^†‡^	<0.001
Serum uric acid (umol/L)	357.56 ± 81.58	352.06 ± 82.19	409.35 ± 79.57^†‡^	395.66 ± 67.89	0.001
Serum urea nitrogen (mmol/L)	6.70 (5.60-8.25)	6.55 (5.02-7.90)	7.30 (5.92-11.10)	9.00(6.87-11.92)^†‡^	<0.001
eGFR (ml/min/1.73m^2^)	79.08 ± 38.25	83.39 ± 34.14	61.07 ± 32.56^‡^	52.34 ± 25.80^†‡^	<0.001
UACR (g/g)	0.29 (0.16-0.57)	1.12 (0.40-1.52)	2.59 (1.14-4.34) ^†‡^	5.61 (4.20-7.70)^†‡#^	<0.001
Hemoglobin (g/L)	137.08 ± 22.29	127.27 ± 19.58	118.35 ± 28.19^†^	109.95 ± 16.93^†‡^	<0.001
Serum albumin (g/L)	43.60 ± 3.89	40.78 ± 6.53	37.08 ± 5.85^†‡^	31.34 ± 4.43^†‡#^	<0.001
Total cholesterol (mmol/L)	4.79 ± 0.91	4.82 ± 1.34	5.43 ± 1.66	6.26 ± 1.48^†‡#^	<0.001
Triglycerides (mmol/L)	2.06 (1.40-2.95)	1.68 (1.10-2.57)	1.99 (1.41-2.66)	1.88(1.35-3.33)	0.307
Serum calcium (mmol/L)	2.34 ± 0.12	2.26 ± 0.15	2.17 ± 0.12^†‡^	2.10 ± 0.14^†‡^	<0.001
Serum phosphorus (mmol/L)	1.14 ± 0.19	1.16 ± 0.26	1.17 ± 0.18	1.17 ± 0.26^‡^	0.920
Parathyroid hormone (pg/mL)	31.75 (24.50-43.93)	32.16 (25.11-44.70)	50.04 (33.17-69.87)^†‡^	45.78 (28.13-76.15)^‡^	<0.001

RAAS, renin angiotensin aldosterone system; HbA1c, glycosylated hemoglobin; eGFR, estimated glomerular filtration rate; UACR, urinary albumin creatinine ratio. P<0.05 was considered statistically significant. † P<0.05 versus the normal group. ‡ P<0.05 versus the mild group. # P<0.05 versus the moderate group. For continuous variables, those with a normal and skewed distribution are described using the mean and standard deviation (SD) and median and interquartile ranges (Q1-Q3), respectively. Categorical variables are presented as frequencies (percentages). Differences in means for quantitative variables were evaluated using ANOVA or the Kruskal-Wallis H test, as appropriate. Categorical variables were compared with the chi-squared test.

### Baseline Histological Characteristics

Regarding the pathological characteristics of DN, we found that the severe group had more serious pathological injury. Patients with lower 25(OH)D levels had more serious glomerular lesions (glomerular classification III-IV) and increased interstitial fibrosis and tubular atrophy (IFTA) scores (score 2-3). In the severe group, glomerular classification III-IV and IFTA score 2-3 account for the vast majority of patients with biopsy-proven DN. The pathological characteristics of patients with different serum 25(OH)D levels are presented in **Table 2**.

**Table 2:** Baseline pathological characteristics of patients with diabetic nephropathy grouped by 25-hydroxyvitamin D level.

Variables	Normal group >20ng/ml (n=25)	Mild group 10-20ng/ml (n=49)	Moderate group 5-10ng/ml (n=49)	Severe group <5ng/ml (n=38)	P value
Glomerular classification					0.020
I-IIa	16 (64.0%)	21 (42.9%)	13 (26.5%)	8 (21.1%)	
IIb	1 (4.0%)	1 (2.0%)	6 (12.2%)	1 (2.6%)	
III	5 (20.0%)	23 (46.9%)	23 (46.9%)	23 (60.5%)	
IV	3 (12.0%)	4 (8.2%)	7 (14.3%)	6 (15.8%)	
IFTA					0.018
0-1	19 (76.0%)	24 (48.9%)	18 (36.7%)	12 (31.6%)	
2	5 (20.0%)	17 (34.7%)	26 (53.1%)	17 (44.7%)	
3	1 (4.0%)	8 (16.3%)	5 (10.2%)	9 (23.7%)	
Interstitial inflammation					0.485
0-1	17 (68.0%)	36 (73.5%)	35 (71.4%)	21 (55.3%)	
2	8 (32.0%)	13 (26.5%)	14 (28.6%)	17 (44.7%)	
Arteriolar hyalinosis					0.764
0	3 (12.0%)	10 (20.4%)	7 (14.3%)	5 (13.2%)	
1	8 (32.0%)	10 (20.4%)	14 (28.6%)	7 (18.4%)	
2	14 (56.0%)	29 (59.2%)	28 (57.1%)	26 (68.4%)	
Arteriosclerosis					0.367
0	7 (28.0%)	11 (22.4%)	8 (16.3%)	8 (21.1%)	
1	9 (36.0%)	24 (49.0%)	32 (65.3%)	19 (50.0%)	
2	9 (36.0%)	14 (28.6%)	9 (18.4%)	11 (28.9%)	

IFTA, interstitial fibrosis and tubular atrophy. P<0.05 was considered statistically significant. Categorical variables are presented as frequencies (percentages), which were compared with the chi-squared test.

### Correlation Between 25-Hydroxyvitamin D Level and Clinicopathological Factors

The correlations between 25(OH)D levels and clinicopathological factors of DN patients are shown in **Table 3**. The 25(OH)D level showed a significant positive correlation with the eGFR (p<0.001), calcium (p<0.001), and hemoglobin (p<0.001) as well as a slightly stronger positive correlation with albumin (p<0.001). In addition, the 25(OH)D level had a strong negative correlation with the UACR (p<0.001) and a weak inverse correlation with creatinine (p<0.001), uric acid (p=0.014), DR (p=0.001), glomerular class (p<0.001) and IFTA (p=0.001).

**Table 3:** Correlation between 25-hydroxyvitamin D level and clinicopathological parameters in diabetic nephropathy.

	Variables	Correlation coefficient (r)	P value
25-hydroxyvitamin D	Clinical findings		
	eGFR	0.380	<0.001
	UACR	-0.762	<0.001
	Creatinine	-0.424	<0.001
	Uric acid	-0.194	0.014
	Albumin	0.655	<0.001
	Hemoglobin	0.402	<0.001
	Calcium	0.522	<0.001
	Diabetic retinopathy	-0.359	0.001
	Total cholesterol	-0.403	<0.001
	Parathyroid hormone	-0.327	<0.001
	urea nitrogen	-0.340	<0.001
	Pathological features		
	Glomerular class	-0.217	<0.001
	IFTA	-0.208	0.001

eGFR, estimated glomerular filtration rate; UACR, urinary microalbumin creatinine ratio; IFTA, interstitial fibrosis and tubular atrophy. P<0.05 was considered statistically significant. Correlations of the 25(OH)D level with clinicopathological findings were analyzed by Spearman’s correlation analysis and Kendall’s tau-b analysis.

### 25-Hydroxyvitamin D Level and Renal Outcomes

**Figure 2:**
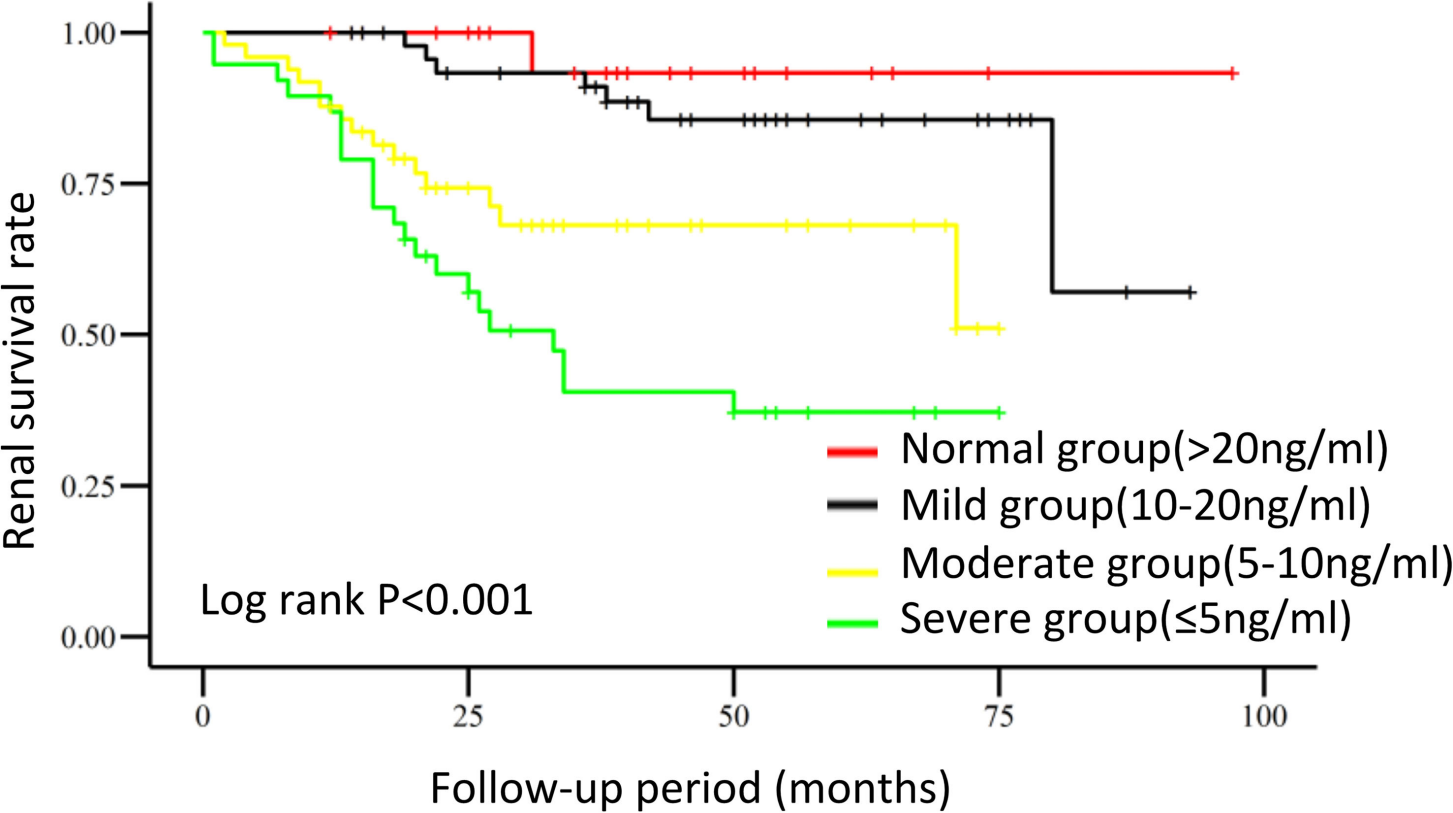
Renal survival in DN patients with different 25-hydroxyvitamin D levels. Kaplan–Meier curves were used to analyze cumulative renal survival, with comparisons between groups being made by the log-rank test.

Survival curves of the renal endpoints are presented in **Figure 2**. The Kaplan–Meier survival analysis indicated that renal survival significantly deteriorated as the level of 25(OH)D decreased (log-rank test, P<0.001). Univariate Cox regression analysis showed that 25(OH)D could significantly impact the renal outcome in these patients with biopsy-proven DN [HR, per SD 25(OH)D 0.261, 95% CI 0.155-0.441, p<0.001]. As the 25(OH)D level decreased, the risk of renal endpoints (double creatinine, ESRD or first onset of dialysis and death) increased; the HRs were 2.921(0.359-23.798, p=0.317) for the mild group, 10.597 (1.383-81.179, p=0.023) for the moderate group, and 20.731 (2.760-155.704, p=0.003) for the severe group, compared to the normal group. In model 3, after adjusting for clinical variables, including age, sex, DR, RAAS, eGFR, UACR, hypertension, cholesterol, triglycerides, duration of DM and pathological characteristics, a lower 25(OH)D level remained a risk factor for renal outcomes. The HRs were 3.446 (95% CI 0.366-32.406, p=0.279) for the mild group, 8.009 (95% CI 0.791-81.102, p=0.078) for the moderate group, and 14.957(95%CI 1.364-163.995, P=0.027) for the severe group. These results are shown in **Table 4** and indicate that the level of 25(OH)D was an independent protective factor for renal endpoints either in univariate analysis or multivariate analysis.

**Table 4:** Association between 25-hydroxyvitamin D and renal outcomes.

		Hazard ratios (95% confidence interval) & p value			
	25(OHD), median (IQR)(g/L)	Unadjusted	Model 1^a^	Model 2^b^	Model 3^c^
Per 1 SD 25(OHD)	9.12 (5.13,15.21)	0.261 (0.155,0.441)	0.301 (0.163,0.554)	0.398 (0.208,0.761)	0.369 ( 0.184,0.741)
		P<0.001	P<0.001	P = 0.005	P = 0.005
Normal group(>20ng/ml)	24.01 (21.17,26.80)	Reference	Reference	Reference	Reference
Mild group(10-20ng/ml)	13.60 (11.81,16.28)	2.921 (0.359,23.798)	3.432 (0.394,29.919)	4.012 (0.453,35.515)	3.446 (0.366,32.406)
		P = 0.317	P = 0.264	P = 0.212	P = 0.279
Moderate group(5-10ng/ml)	7.75 (6.15,8.85)	10.597 (1.383,81.179)	9.092 (1.120,73.834)	8.482 (0.925,77.796)	8.009 (0.791,81.102)
		P = 0.023	P = 0.039	P = 0.059	P = 0.078
Severe group(≤5ng/ml)	3.24 (2.00,3.93)	20.731(2.760,155.704)	18.451 (2.181,156.091)	14.208 (1.456,138.664)	14.957 (1.364,163.995)
		P = 0.003	P = 0.007	P = 0.022	P = 0.027

Cox analysis was used to explore the relationships between 25(OH)D levels and renal outcomes. 25(OH)D was analyzed as a continuous variable with hazard ratios (HRs) calculated per SD increment. SD: standard deviation.

a Model 1 adjusted for age, sex, DR, hypertension, cholesterol, triglycerides, duration of DM and use of RAAS;

b Model 2 adjusted for covariates in model 1 plus eGFR、UACR;

c Model 3 adjusted for covariates in model 2 plus renal pathological findings (the glomerular class, IFTA, interstitial inflammation scores, arteriolar hyalinosis).

## Discussion

In this study, we assessed the relationship of the 25(OH)D level with the clinical characteristics and renal survival of 161 type 2 DM patients with biopsy-proven DN. Our study showed that the 25(OH)D level had a significant positive correlation with eGFR, albumin, and calcium and a negative correlation with the UACR, creatinine, uric acid, PTH, etc. The results also demonstrated that DN patients with lower levels of 25(OH)D were more prone to progress to renal endpoints, which was independent of age, sex, course of diabetes, DR, creatinine, UACR, eGFR and pathological damage. Clinicians often pay more attention to the impact of proteinuria and renal function on DN but ignore some other indicators. Recently, studies have shown that 25(OH)D deficiency is a prominent feature in patients with CKD and even proved the protective role of 25(OH)D in patients with DN (22, 23). Xiao X et al. found that 25(OH)D was significantly positively correlated with eGFR and negatively correlated with the UACR (24). A double-blind, randomized, placebo-controlled trial of 155 DN patients demonstrated that 25(OH)D can reduce proteinuria and serum creatinine by inhibiting the RAAS (25). In agreement with all these studies, we suggested that patients with severe 25(OH)D deficiency should be followed up carefully and given active treatment.

Our study is the first to explore the relationship between renal pathological classification and 25(OH)D levels in patients with biopsy-proven DN. We found that the 25(OH)D level had a significant correlation with pathological features. The lower the 25(OH)D level is, the more serious the glomeruli damage is, exhibiting corresponding degrees of mesangial expansion, Kimmelstiel-Wilson (K-W) nodules and glomerular sclerosis. The IFTA score, which reflects the extent of tubulointerstitial injury, increased as the level of 25(OH)D decreased. These results all suggest that the level of 25(OH)D has a close association with the severity of renal pathological damage. The diagnosis of DN is traditionally dependent on clinical manifestations rather than renal pathology. Considering that NDRD is common in diabetes with renal biopsy (26–28), the results of these studies might be less convincing. Therefore, the finding in our study from biopsy-proven DN patients could be more reliable.

In consist with previous studies, we confirmed that 25(OH)D is associated with the serum level of calcium (29) and PTH (30). Low level of 25(OH)D is usually related to the clinical symptoms of vitamin D deficiency (31). In addition to the regulation of bone metabolism, calcium and phosphorus metabolism, the effects of 25(OH)D on the cell cycle, immune regulation, maintenance of neuromuscular function and inhibition of the production of inflammatory factors have attracted increasing attention in recent years (6, 8, 32). A lower level of 25(OH)D might be caused by ineffective synthesis in skin upon exposure to ultraviolet B radiation and reduced nutritional intake (33). The deregulation of Fibroblast growth factor 23 (FGF23), an essential regulator to maintain phosphate and calcium homeostasis (34), could be another important reason to suppress the conversion of inactive 25(OH)D into active 1,25(OH)_2_D in diabetic patients (35). In our study, we also demonstrated that low level of 25(OH)D is an important risk factor for massive proteinuria and renal function decline. The level of 25(OH)D was correlated with renal function, UACR and glomerular pathological classification, which may provide some explanation for the association between 25(OH)D deficiency and renal outcome in DN patients. In addition, we also found that 25(OH)D levels were negatively correlated with the severity of DR in DN patients, which is consistent with other clinical studies (36, 37). The mechanism is probably due to the expression of the vitamin D receptor in the human retina (38). Thus, early detection of the 25(OH)D level is also very important for the prevention of DR.

How 25(OH)D exerts a protective role in diabetic kidney disease is still unclear. Studies have shown that 25(OH)D can ameliorate glomerular and tubular injury in murine models of kidney diseases. For example, 25(OH)D attenuated podocyte loss and apoptosis and reduced glomerular fibrosis in diabetic mice (39). In the CKD model of 5/6 nephrectomy rats, the vitamin D analog paricacitol could reduce proteinuria and protect glomeruli and tubules from injury by inhibiting the activation of RAAS in the kidney (40). In experimental models, the use of vitamin D analogs to block RAAS activation exerts a therapeutic effect by enhancing the action of RAAS blockers (41). Lower 25(OH)D levels are particularly pernicious in the setting of RAAS activation and hyperfiltration in diabetic mice (42). Nakai et al. (43) showed that the vitamin D analog masalone can reduce the oxidative stress response through the Nrf2-Keap1 pathway to delay the progression of DN in diabetic rats. In another clinical study of a group of DN patients, the expression of IL-17, IL-6, IL-1 and TNF-α was found to be significantly decreased by adding paricalcitol, suggesting the immune regulation and anti-inflammatory effect of 25(OH)D (44). All these findings demonstrated that 25(OH)D can ameliorate glomerular and tubular injury and that improving the level of 25(OH)D is beneficial to the treatment of DN. However, the underlying mechanism of renal protection of 25(OH)D needs to be further clarified.

There are still some limitations of this study. Firstly, this is a retrospective study with the inevitable bias of choice. Secondly, the sample size of DN patients was relatively small. Multicenter and prospective studies can further explore the relationship between 25(OH)D and DN prognosis, and sample validation from other research centers is required. Finally, some potential confounders, such as outdoor exercise, sunshine exposure, nutritional status, smoking status, and season of 25(OH)D measurement, were not included in the analysis.

## Conclusions

In conclusion, our study is the first to explore the association of 25(OH)D levels and renal pathological findings in patients with biopsy-proven DN. We found that a 25(OH)D level less than 5 ng/ml was correlated with worse renal function, more severe glomerular and tubular pathological injury scores and poorer renal prognosis in patients with biopsy-proven DN. These new findings will provide potential management strategies that may improve renal outcomes in DN patients. Further clinical studies are needed to assess whether additional supplementation with 25(OH)D could improve renal survival in these patients.

## Data Availability Statement

The raw data supporting the conclusions of this article will be made available by the authors, without undue reservation.

## Ethics Statement

The studies involving human participants were reviewed and approved by the Ethics Committee of the Shanghai Jiao Tong University Affiliated Sixth People’s Hospital. The patients/participants provided their written informed consent to participate in this study.

## Author Contributions

In the study, YF, QJ, and NW designed the research project. TZ, LS, ZL, QJ, JJ, and HX performed the study, collected the clinical data and worked on the follow-up of patients. TZ and LS analyzed the data. TZ and LS drafted the manuscript. YF and QJ revised and approved the final version of the manuscript.

## Funding

This study was supported by National Nature Science Foundation of China (82170727, 81870504, and 81870468), the Shanghai Jiao Tong University Gaofeng Talent Training Plan and a clinical project (20192833), Open Project of Shanghai Key Laboratory of Sleep Disordered Breathing (SHKSDB-KF-19-04), Three-year Project of Shanghai TCM Development (ZT(2018-2020)-FWTX-2003), Star Program of Shanghai Jiao Tong University (20190102), Open project of National Science and Technology Infrastructure of translational medicine (Shanghai, TMSK-2021-109)

## Conflict of Interest

The authors declare that the research was conducted in the absence of any commercial or financial relationships that could be construed as a potential conflict of interest.

## Publisher’s Note

All claims expressed in this article are solely those of the authors and do not necessarily represent those of their affiliated organizations, or those of the publisher, the editors and the reviewers. Any product that may be evaluated in this article, or claim that may be made by its manufacturer, is not guaranteed or endorsed by the publisher.
